# The Japanese Breast Cancer Society Clinical Practice Guidelines for Breast Cancer, 2022 Edition: changes from the 2018 edition and general statements on breast cancer treatment

**DOI:** 10.1007/s12282-024-01566-6

**Published:** 2024-04-03

**Authors:** Yutaka Yamamoto, Chikako Yamauchi, Tatsuya Toyama, Shigenori Nagai, Takehiko Sakai, Goro Kutomi, Michio Yoshimura, Masaaki Kawai, Shoichiro Ohtani, Kazunori Kubota, Kazutaka Nakashima, Naoko Honma, Masayuki Yoshida, Eriko Tokunaga, Naruto Taira, Hiroji Iwata, Shigehira Saji

**Affiliations:** 1 Department of Breast and Endocrine Surgery, Kumamoto University Hospital, 1-1-1 Honjo, Chuo-Ku, Kumamoto, 860-8556 Japan; 2 Department of Radiation Oncology, Shiga General Hospital, Shiga, Japan; 3 Department of Breast Surgery, Nagoya City University, Aichi, Japan; 4 Division of Breast Oncology, Saitama Cancer Center, Saitama, Japan; 5 Department of Breast Surgical Oncology, Cancer Institute Hospital, Japanese Foundation for Cancer Research, Tokyo, Japan; 6 Department of Surgery, Surgical Oncology and Science, Sapporo Medical University Hospital, Sapporo, Japan; 7 Department of Radiation Oncology and Image-Applied Therapy, Kyoto University Graduate School of Medicine, Kyoto, Japan; 8 Department of Surgery I, Yamagata University Hospital, Yamagata, Japan; 9 Ohtani Shoichiro Breast Clinic, Hiroshima, Japan; 10 Department of Radiology, Dokkyo Medical University Saitama Medical Center, Saitama, Japan; 11 Department of General Surgery, Kawasaki Medical School General Medical Center, Okayama, Japan; 12 Department of Pathology, Faculty of Medicine, Toho University, Tokyo, Japan; 13 Department of Diagnostic Pathology, National Cancer Center Hospital, Tokyo, Japan; 14 Department of Breast Oncology, NHO Kyushu Cancer Center, Fukuoka, Japan; 15 Department of Breast and Thyroid Surgery, Kawasaki Medical School Hospital, Kurashiki, Japan; 16 Department of Breast Oncology, Aichi Cancer Center, Nagoya, Japan; 17 Department of Medical Oncology, School of Medicine, Fukushima Medical University, Fukushima, Japan

**Keywords:** Breast cancer, Guideline, Local therapy, Systemic therapy

## Abstract

The Japanese Breast Cancer Society Clinical Practice Guidelines for Breast Cancer, 2022 Edition was published in June 2022. The guidelines were prepared while conforming as much as possible to the “Minds Manual for Guideline Development 2020 ver. 3.0.” edited by the Minds Manual Development Committee of the Japan Council for Quality Health Care in 2021. In addition, a survey of Japanese Breast Cancer Society members on the 2018 edition of the guidelines was conducted from February 19 to March 4, 2021. Based on the responses from over 600 members, original innovations were made to make the guidelines more user-friendly. The 2018 edition of the guidelines was developed to provide support tools for physicians and patients to utilize shared decision-making. The 2022 guidelines consist of two volumes: (1) an “Epidemiology and Diagnosis” section covering “Screening and Diagnosis”, “Radiological diagnosis”, and “Pathological diagnosis”, and (2) a “Treatment” section covering “Surgical therapy”, “Radiation therapy”, and “Systemic therapy”. We believe that this concise summary of the guidelines will be useful to physicians and researchers in Japan and overseas.

## Introduction

The “Science-Based Clinical Practice Guidelines for Breast Cancer” was developed as a research report in 2002 with a grant from the Ministry of Health, Labor and Welfare. This report marked the beginning of the current breast cancer treatment guidelines. Subsequently, development of the guidelines was transferred to the Japanese Breast Cancer Society (JBCS). Up to the 2015 edition, this process has produced highly regarded complete guidelines that have contributed significantly to standardization of breast cancer treatment and care in Japan. However, the balance of benefits and harms was insufficiently examined in the guidelines up to the 2015 edition [1]. Thus, the method used to create the 2018 edition of the guidelines [2] was significantly changed. The 2022 edition follows this method, while also reflecting the results of a survey of JBCS members and suggestions from solicitation of public comments.

## Concepts and methods of development of the 2018 and 2022 JBCS guidelines

The 2018 guidelines [2] were designed to provide support tools for physicians and patients to utilize shared decision-making, and were developed in accordance with the “Minds Manual for Guideline Development 2014” [3]. Briefly, for each clinical question (CQ), multiple outcomes (about three to six, both beneficial and adverse) and a clinical importance level (1–9) were determined for each outcome. After a literature search using keywords related to the CQs, a quantitative or qualitative systematic review was conducted for each outcome, and the strength of recommendation for each CQ was discussed at board meetings in terms of the balance between benefit and harm. The final recommendations in each session were discussed and voted on at a decision meeting attended by physicians, nurses, pharmacists, and patients. Based on these decisions, the responsible committee members wrote commentaries and confirmed these with each other to produce the final version.

The 2022 edition was based on the concepts and methods used in the 2018 edition, while conforming as much as possible to the “Minds Manual for Guideline Development 2020 ver. 3.0.” [4]. In addition, a JBCS member survey on the 2018 guidelines was conducted from February 19 to March 4, 2021. Changes to make the guidelines more user-friendly were then made based on more than 600 responses to the survey from JBCS members.

The 2022 guidelines consist of two volumes: (1) an “Epidemiology and Diagnosis” section covering “Screening and Diagnosis [5]”, “Radiological diagnosis [6]”, and “Pathological diagnosis [7]”, and (2) a “Treatment” section covering “Surgical therapy [8]”, “Radiation therapy [9]”, and “Systemic therapy []

## Structure and development of the 2022 guidelines

A new chairperson and committee members were appointed for this revision and a kick-off meeting was held in October 2020. After revising the web version of the previous edition, a plenary committee meeting was held in February 2021 and development of the 2022 edition was initiated.

### Structure of the guidelines

General statements: The guidelines describe the basic concepts and flow of treatment, definitions of terms, historical progress, and the minimum necessary textbook knowledge. In the treatment section, a flowchart with links to each CQ is included as a “Treatment” (Surgical therapy, Radiation therapy, and Systemic therapy) overview.

BQ (background question): A question on standard treatment that must be performed or on widely practiced treatment, but for which no new data that would strengthen the rationale are available.

CQ (clinical question): A difficult issue in daily clinical practice is identified, a quantitative or qualitative systematic review is conducted, and a recommendation and strength of the recommendation are determined through a vote at a recommendation meeting.

FRQ (future research question): This section explains the current thinking on CQs that are considered to be important future issues, but for which there is insufficient data to address the issue as a CQ, and those for which new data are expected to be generated.

### Strength of recommendation, grade of evidence, strength of evidence, consensus rate, and recommendation points

Recommendation grades are shown in Table 1. These grades were determined based on the balance of risks and benefits of the intervention in routine clinical practice, consistency with patient preferences, and economic perspectives. The strength of the recommendation follows the “Minds Manual for Guideline Development 2020 ver 3.0.” [4] and is divided into four grades. Most of the CQs for epidemiology and prevention are not for interventions, but for issues to be aware of in daily life. Therefore, we did not take a position of recommending use or non-use of an intervention, but rather we stated the strength of the scientific evidence as an evidence grade (Table 2). The “strength of evidence” is indicated in the recommendation text as “strong,” “medium,” “weak,” or “very weak” on a four-point scale (Table 3), with stronger overall evidence tending to make the recommendation “stronger” for all outcomes for each CQ. However, there are cases in which the recommendation is strongly recommended even if the strength of evidence is “medium” and cases in which the recommendation is weak even if the strength of evidence is “strong”.

**Table 1:** Strength of recommendations

Strength of recommendation	Statement	Clinical implications
1	Strongly recommended	Strongly recommended. (If the CQ is targeted, it will be performed in 90% of patients. If the target is broad, it will be performed in about 70–80% of patients)
2	Weakly recommended	Not mandatory, but recommended based on the balance of benefits and harms and the patient’s sense of values, and after consultation at the site. (If the CQ is targeted, it will be performed in more than 50% of patients. If the target is broad, it will be performed in about 30–40% of patients)
3	Weakly not recommended	The opposite of a weak recommendation. Given the balance of benefits and harms and the values of the patient, the recommendation is not to perform the procedure. (If the CQ is targeted, it will not be performed in more than 50% of patients. If the target is broad, it will not be performed in about 30 to 40% of patients)
4	Strongly not recommended	The harms greatly outweigh the benefits, and it is strongly recommended that this is not undertaken. (If the CQ is targeted, it will not be performed in 90% of patients. If the target is broad, it will not be performed in about 70 to 80% of patients)

The information in parentheses is provided to give a sense of the strengths and weaknesses noted in the decision-making meeting

*CQ* clinical question

**Table 2:** Evidence grades [2]

Convincing	There is enough evidence to determine that an association with cancer risk is certain and taking preventive action is recommended
Probable	There is enough evidence to determine that an association with cancer risk is almost certain and taking preventive action is generally recommended
Limited—suggestive	Neither convincing nor probable can be determined, but there is evidence suggesting an association with cancer risk
Limited—no conclusion	Data are insufficient and an association with cancer risk cannot be determined
Substantial effect on risk unlikely	There is enough evidence to determine that there is no substantial effect on cancer risk

**Table 3:** Certainty of overall outcome evidence for recommendation decisions (strength of evidence)

A (Strong)	Strong confidence in the adequacy of the effect estimates to support the recommendation
B (Moderate)	Moderately confidence in the adequacy of the effect estimates to support the recommendation
C (Weak)	Limited confidence in the adequacy of the effect estimates to support the recommendation
D (Very weak)	Little confidence in the adequacy of the effect estimates to support the recommendation

Starting with the 2022 edition, a “Key Points in Recommendations” section appears below the CQ and recommendation text. This section states the conditions, information, and points to note for understanding the recommendation text. At the end of each CQ, details of the voting results at the decision meeting are also provided.

## General statements

### Ductal carcinoma in situ (TisN0M Stage 0)

Definition: Breast cancer cells remain in the ducts; also known as “intraductal carcinoma”.

**Fig. 1:**
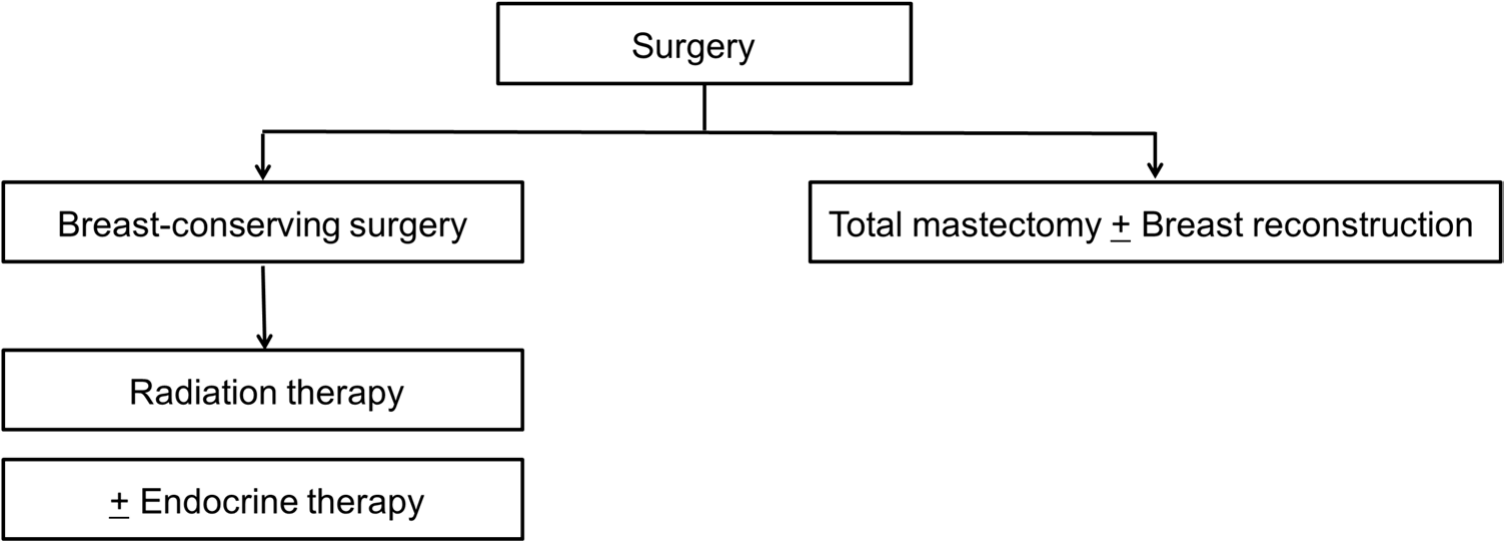
Treatment strategy for ductal carcinoma in situ

Treatment strategies for ductal carcinoma in situ (DCIS) (Fig. 1):

1. Local therapy: The main treatment for DCIS is surgery and/or radiation therapy.
2. Systemic therapy: In a case of hormone receptor-positive DCIS, endocrine therapy is an option to suppress ipsilateral breast cancer recurrence, but not affect distant metastases.

### Early breast cancer (stage I to IIIA)

Purpose of treatment: Treatment for early breast cancer (EBC) includes local therapy (surgery and/or radiation therapy) for the primary tumor and/or axillary lymph node in which cancer is thought to be present based on preoperative diagnosis, and systemic treatment to eradicate and control potential micrometastases. The goals are to achieve a cure and prolong survival.

**Fig. 2:**
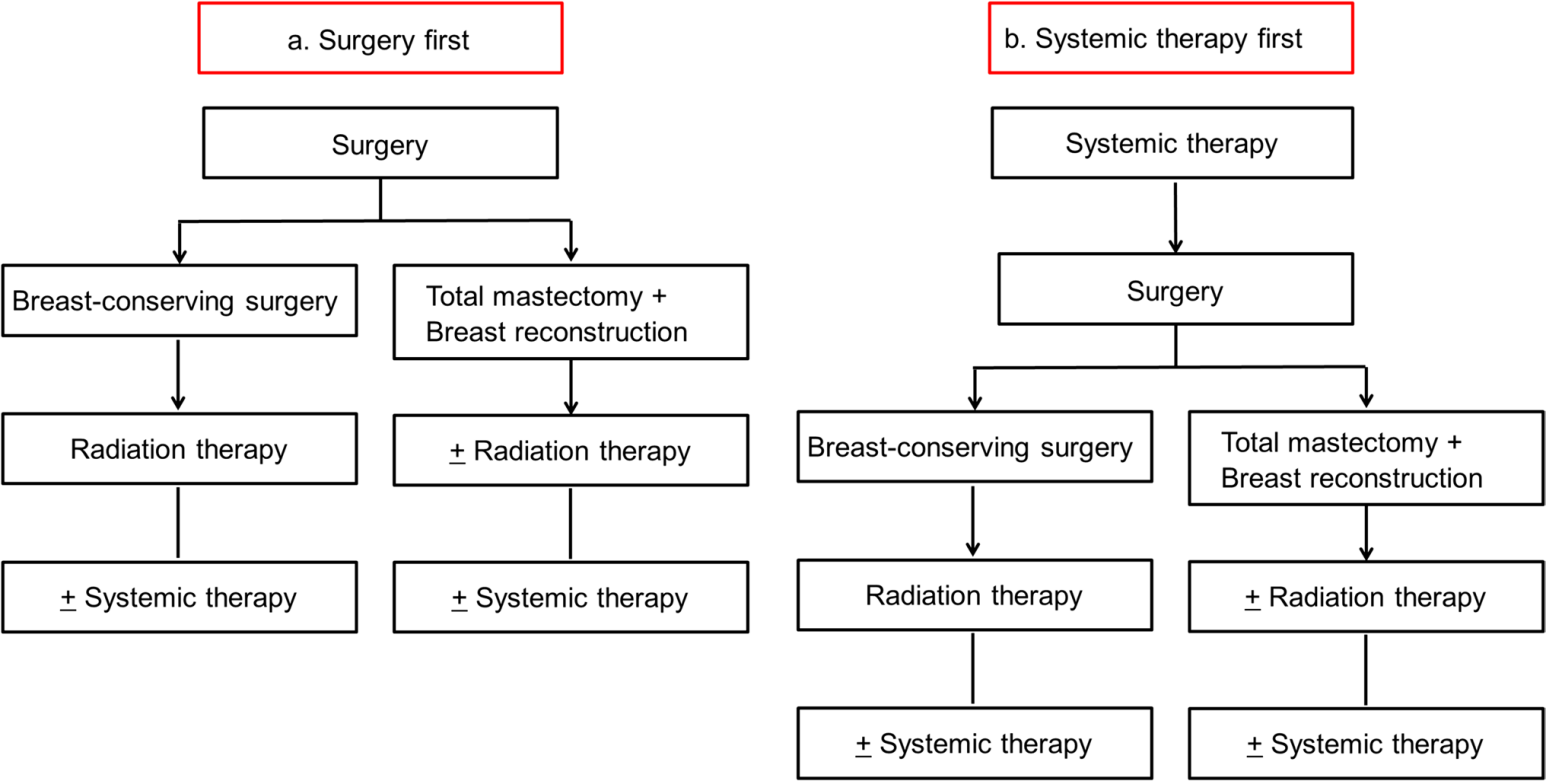
Treatment strategy for early breast cancer

Treatment strategy (Fig. 2): The treatment strategy including local and systemic therapy for EBC is determined based on a comprehensive evaluation of predictive factors, such as hormone receptor and HER2 status, and prognostic factors, such as clinical stage and histological grade.

### Locally advanced breast cancer (stage IIIb or IIIc)

Definition: Locally advanced breast cancer (LABC) refers to inoperable locally advanced cancer without distant metastases (Stage IIIb or IIIc).

**Fig. 3:**
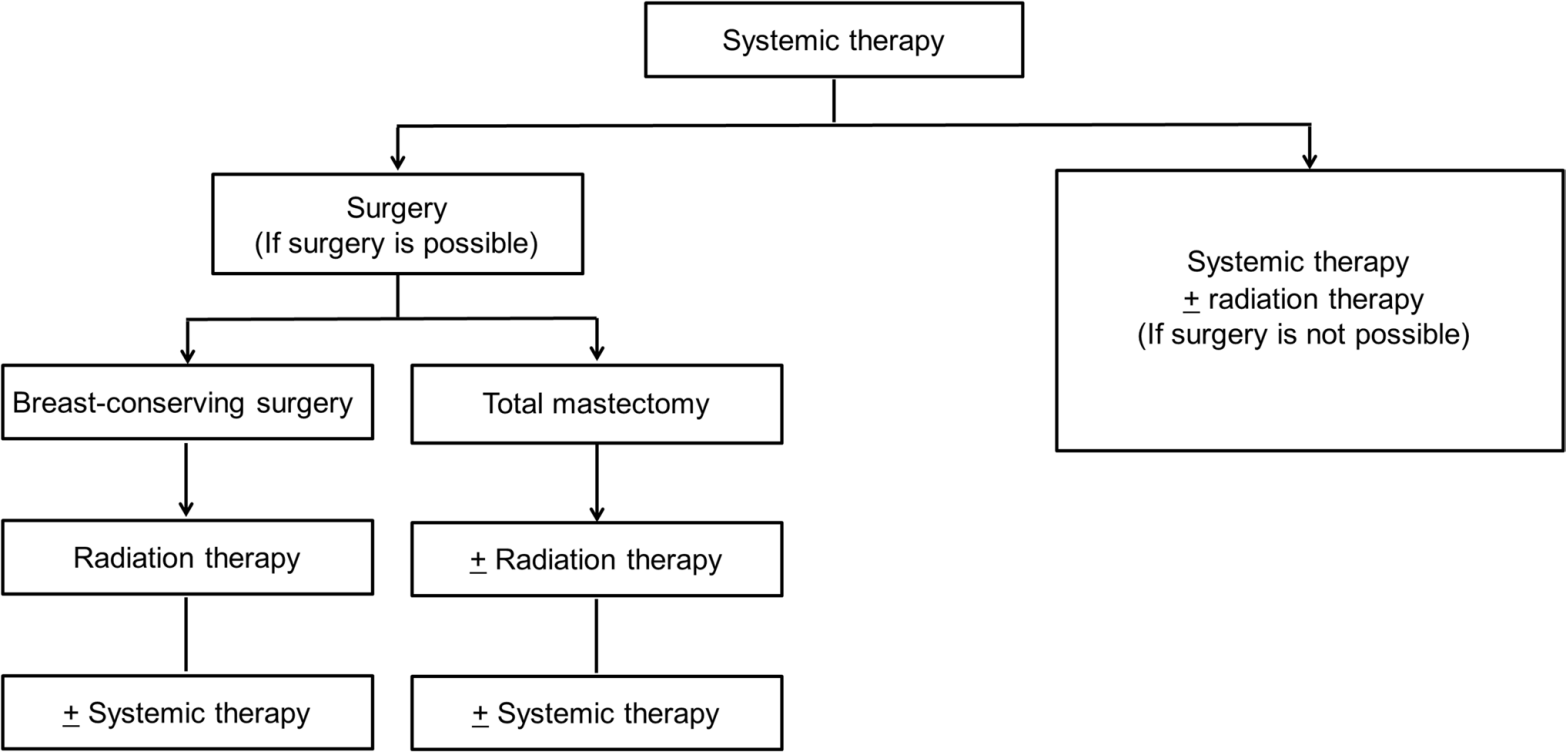
Treatment strategy for locally advanced breast cancer

Treatment strategy (Fig. 3): The goal is to cure the disease using a multidisciplinary approach including systemic and local therapies. Standard treatment for LABC is initial systemic therapy including chemotherapy, followed by surgery and radiation therapy.

### Metastatic breast cancer (stage IV or distant metastatic recurrence)

Purpose of treatment: Treatment for metastatic breast cancer (MBC) is provided to palliate symptoms, prevent development of symptoms and prolong survival.

Treatment strategy: Factors to consider in treatment selection include patient factors such as age, comorbidities and socioeconomic context; tumor factors such as biological characteristics, metastatic sites, disease-free interval, neoadjuvant regimens and/or systemic treatments, symptoms caused by the tumor, and clinical evidence; and patient preference. MBC is primarily systemic, but treatment strategies vary by tumor subtype.

**Fig. 4:**
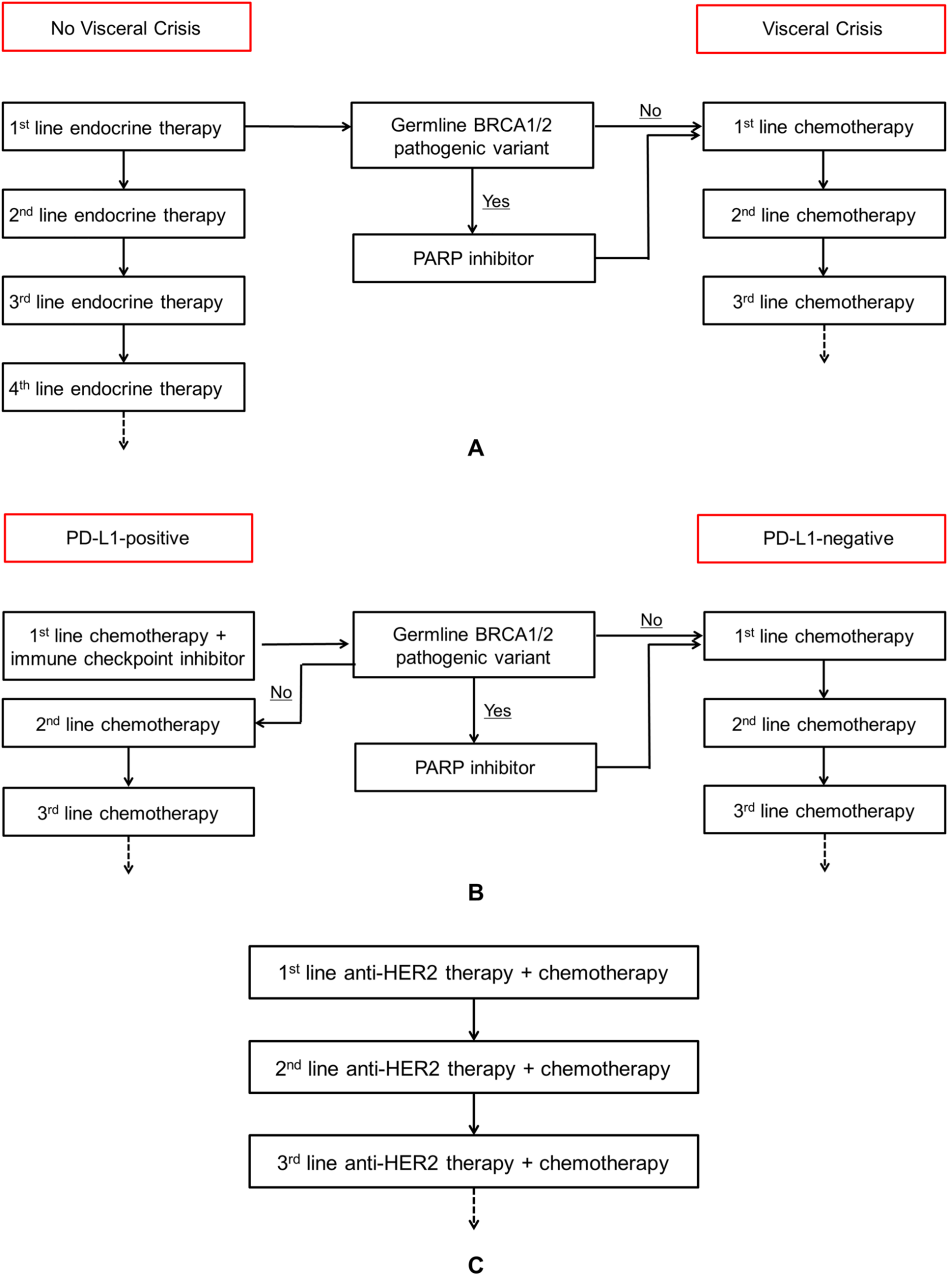
Treatment strategies for **a** hormone receptor-positive/HER2-negative metastatic breast cancer, **b** triple-negative metastatic breast cancer, and **c** HER2-positive metastatic breast cancer

Hormone receptor-positive/HER2-negative MBC (Fig. 4a): In the absence of a visceral crisis, the initial treatment should be endocrine therapy plus a cyclin-dependent kinase (CDK)4/6 inhibitor or endocrine monotherapy. If the tumor responds to first-line endocrine therapy, this therapy should be continued until ineffective. If endocrine therapy fails or in a case with visceral crisis, treatment should be switched to PARP inhibitor if a germline *BRCA1/2* mutation is present or chemotherapy.

Triple-negative MBC (Fig. 4b): In PD-L1-positive disease, an immune checkpoint inhibitor (ICI) plus chemotherapy is recommended as first-line therapy. If a germline *BRCA1/2* mutation is present, use of a PARP inhibitor is recommended after or before ICI plus chemotherapy. In PD-L1-negative disease with a germline *BRCA1/2* mutation, a PARP inhibitor is recommended as first-line treatment. In PD-L1-negative disease without a germline *BRCA1/2* mutation, chemotherapy is recommended as first-line treatment.

HER2-positive MBC (Fig. 4c): Anti-HER2 therapy in combination with chemotherapy is the basic approach. Trastuzumab and pertuzumab with chemotherapy is recommended as first-line treatment. Trastuzumab deruxtecan is recommended as second-line treatment.

## Conclusion

Daily practice is a series of interventions (diagnosis, surgery, radiation therapy, systemic therapy, etc.) and it is important to consider the benefits and harms when deciding which measures to take. We believe that the 2022 guidelines provide both a guide to standard practice and a repository of accurate information. However, the patient’s situation may not allow a standard choice recommended by the guidelines. In such cases, shared decision-making, in which the next intervention is determined based on shared knowledge and understanding, is important in building mutually trusting relationships between physicians and patients. We hope that the JBCS Clinical Practice Guidelines will be used as a tool for medical practitioners to walk alongside their patients in breast cancer care. We also believe that this summary of the English version of the 2022 Breast Cancer Treatment Guidelines will be useful for researchers in Japan and overseas.

## Data Availability

The raw data reauired to reproduce the abouve findings are available to downlaoad from https://jbcs.xsrv.jp/guideline/2022/.
