# Correction: The Japanese Breast Cancer Society Clinical Practice Guidelines for Breast Cancer, 2022 Edition: changes from the 2018 edition and general statements on breast cancer treatment

**DOI:** 10.1007/s12282-024-01589-z

**Published:** 2024-05-12

**Authors:** Yutaka Yamamoto, Chikako Yamauchi, Tatsuya Toyama, Shigenori Nagai, Takehiko Sakai, Goro Kutomi, Michio Yoshimura, Masaaki Kawai, Shoichiro Ohtani, Kazunori Kubota, Kazutaka Nakashima, Naoko Honma, Masayuki Yoshida, Eriko Tokunaga, Naruto Taira, Hiroji Iwata, Shigehira Saji

**Affiliations:** 1 Department of Breast and Endocrine Surgery, Kumamoto University Hospital, 1-1-1 Honjo, Chuo-Ku, Kumamoto, 860-8556 Japan; 2 Department of Radiation Oncology, Shiga General Hospital, Shiga, Japan; 3 Department of Breast Surgery, Nagoya City University, Aichi, Japan; 4 Division of Breast Oncology, Saitama Cancer Center, Saitama, Japan; 5 Department of Breast Surgical Oncology, Cancer Institute Hospital, Japanese Foundation for Cancer Research, Tokyo, Japan; 6 Department of Surgery, Surgical Oncology and Science, Sapporo Medical University Hospital, Sapporo, Japan; 7 Department of Radiation Oncology and Image-Applied Therapy, Kyoto University Graduate School of Medicine, Kyoto, Japan; 8 Department of Surgery I, Yamagata University Hospital, Yamagata, Japan; 9 Ohtani Shoichiro Breast Clinic, Hiroshima, Japan; 10 Department of Radiology, Dokkyo Medical University Saitama Medical Center, Saitama, Japan; 11 Department of General Surgery, Kawasaki Medical School General Medical Center, Okayama, Japan; 12 Department of Pathology, Faculty of Medicine, Toho University, Tokyo, Japan; 13 Department of Diagnostic Pathology, National Cancer Center Hospital, Tokyo, Japan; 14 Department of Breast Oncology, NHO Kyushu Cancer Center, Fukuoka, Japan; 15 Department of Breast and Thyroid Surgery, Kawasaki Medical School Hospital, Kurashiki, Japan; 16 Department of Breast Oncology, Aichi Cancer Center, Nagoya, Japan; 17 Department of Medical Oncology, School of Medicine, Fukushima Medical University, Fukushima, Japan

**Correction to: Breast Cancer** 10.1007/s12282-024-01566-6

In the sentence beginning 'The 2022 guidelines consist of two volumes…' in this article, the text '[]' should have read '[10]'.

In this article, ref. 4 was missing and should have been 'Minds Manual Developing Committee ed. Minds manual for guideline development 2020 ver 3.0. Tokyo: Igaku Shoin; 2014.'. The subsequent references are renumbered and the complete reference list is given below:

**References**

1. Mukai H, Aihara T, Yamamoto Y, Takahashi M, Toyama T, Sagara Y, Yamaguchi H, Akabane H, Tsurutani J, Hara F, Fujisawa T, Yamamoto N, Ohsumi S, Japanese Breast Cancer Society. The Japanese Breast Cancer Society 2013 clinical practice guidelines: history, policy and mission. Breast Cancer. 2015;22:1–4.

2. Iwata H, Saji S, Ikeda M, Inokuchi M, Uematsu T, Toyama T, Horii R, Yamauchi C. The Japanese Breast Cancer Society Clinical Practice Guidelines, 2018 edition: a tool for shared decision making between doctor and patient. Breast Cancer. 2020;27(1):1–3.

3. Morizane T, Yoshida M, Kojimahara N. Minds Handbook for Clinical Practice Guideline Development 2014. In: Fukui T, Yamaguchi N, editors. Minds Guideline Center, Japan Council for Quality Health Care; 2015.

4. Minds Manual Developing Committee ed. Minds manual for guideline development 2020 ver 3.0. Tokyo: Igaku Shoin; 2014.

5. Kawai M, Ohtani S, Iwasaki M, Yamamoto S, Takamatsu K, Okamura H, Arai M, Nomura T, Ozaki S, Shibata KI, Akabane A, Motoi F, Yamauchi C, Yamamoto Y, Iwata H, Saji S. The Japanese Breast Cancer Society clinical practice guidelines for epidemiology and prevention of breast cancer, 2022 edition. Breast Cancer. 2023. 10.1007/s12282-023-01531-9.

6. Kubota K, Nakashima K, Nakashima K, Kataoka M, Inoue K, Goto M, Kanbayashi C, Hirokaga K, Yamaguchi K, Ohta Y, Suzuki A. The Japanese breast cancer society clinical practice guidelines for breast cancer screening and diagnosis, 2022 edition. Breast Cancer. 2023. 10.1007/s12282-023-01521-x.

7. Honma N, Yoshida M, Kinowaki K, Horii R, Katsurada Y, Murata Y, Shimizu A, Tanabe Y, Yamauchi C, Yamamoto Y, Iwata H, Saji S. The Japanese breast cancer society clinical practice guidelines for pathological diagnosis of breast cancer, 2022 edition. Breast Cancer. 2024;31(1):8–15. 10.1007/s12282-023-01518-6.

8. Sakai T, Kutomi G, Shien T, Asaga S, Aruga T, Ishitobi M, Kuba S, Sawaki M, Terata K, Tomita K, Yamauchi C, Yamamoto Y, Iwata H, Saji S. The Japanese Breast Cancer Society Clinical Practice Guidelines for surgical treatment of breast cancer, 2022 edition. Breast Cancer. 2024;31(1):1–7. 10.1007/s12282-023-01510-0.

9. Yoshimura M, Yamauchi C, Sanuki N, Hamamoto Y, Hirata K, Kawamori J, Kawamura M, Ogita M, Yamamoto Y, Iwata H, Saji S. The Japanese Breast Cancer Society Clinical Practice Guidelines for radiation treatment of breast cancer. Breast Cancer.

10. Terada M, Ito A, Kikawa Y, Koizumi K, Naito Y, Shimoi T, Ishihara M, Yamanaka T, Ozaki Y, Hara F, Nakamura R, Hattori M, Miyashita M, Kondo N, Yoshinami T, Takada M, Matsumoto K, Narui K, Sasada S, Iwamoto T, Hosoda M, Takano Y, Oba T, Sakai H, Murakami A, Higuchi T, Tsuchida J, Tanabe Y, Shigechi T, Tokuda E, Harao M, Kashiwagi S, Mase J, Watanabe J, Nagai SE, Yamauchi C, Yamamoto Y, Iwata H, Saji S, Toyama T. The Japanese Breast Cancer Society Clinical Practice Guidelines for systemic treatment of breast cancer, 2022 edition. Breast Cancer. 2023;30(6):872–84. 10.1007/s12282-023-01505-x.

